# LGR5 regulates pro-survival MEK/ERK and proliferative Wnt/β-catenin signalling in neuroblastoma

**DOI:** 10.18632/oncotarget.5548

**Published:** 2015-10-23

**Authors:** Gabriella Cunha Vieira, S. Chockalingam, Zsombor Melegh, Alexander Greenhough, Sally Malik, Marianna Szemes, Ji Hyun Park, Abderrahmane Kaidi, Li Zhou, Daniel Catchpoole, Rhys Morgan, David O. Bates, Peter J. Gabb, Karim Malik

**Affiliations:** ^1^ Cancer Epigenetics Laboratory and, School of Cellular & Molecular Medicine, University of Bristol, Bristol, UK; ^2^ Department of Cellular Pathology, Southmead Hospital, Bristol, UK; ^3^ Colorectal Cancer Laboratory, School of Cellular & Molecular Medicine, University of Bristol, Bristol, UK; ^4^ The Kids Research Institute, The Children's Hospital at Westmead, Westmead, New South Wales, Australia; ^5^ Cancer Biology, Division of Cancer and Stem Cells, School of Medicine, University of Nottingham, Nottingham, UK

**Keywords:** neuroblastoma, LGR5, Wnt/β-catenin, MEK/ERK, cell survival

## Abstract

LGR5 is a marker of normal and cancer stem cells in various tissues where it functions as a receptor for R-spondins and increases canonical Wnt signalling amplitude. Here we report that LGR5 is also highly expressed in a subset of high grade neuroblastomas. Neuroblastoma is a clinically heterogenous paediatric cancer comprising a high proportion of poor prognosis cases (~40%) which are frequently lethal. Unlike many cancers, Wnt pathway mutations are not apparent in neuroblastoma, although previous microarray analyses have implicated deregulated Wnt signalling in high-risk neuroblastoma. We demonstrate that LGR5 facilitates high Wnt signalling in neuroblastoma cell lines treated with Wnt3a and R-spondins, with SK-N-BE(2)-C, SK-N-NAS and SH-SY5Y cell-lines all displaying strong Wnt induction. These lines represent MYCN-amplified, NRAS and ALK mutant neuroblastoma subtypes respectively. Wnt3a/R-Spondin treatment also promoted nuclear translocation of β-catenin, increased proliferation and activation of Wnt target genes. Strikingly, short-interfering RNA mediated knockdown of LGR5 induces dramatic Wnt-independent apoptosis in all three cell-lines, accompanied by greatly diminished phosphorylation of mitogen/extracellular signal-regulated kinases (MEK1/2) and extracellular signal-regulated kinases (ERK1/2), and an increase of BimEL, an apoptosis facilitator downstream of ERK. Akt signalling is also decreased by a Rictor dependent, PDK1-independent mechanism. LGR5 expression is cell cycle regulated and LGR5 depletion triggers G1 cell-cycle arrest, increased p27 and decreased phosphorylated retinoblastoma protein. Our study therefore characterises new cancer-associated pathways regulated by LGR5, and suggest that targeting of LGR5 may be of therapeutic benefit for neuroblastomas with diverse etiologies, as well as other cancers expressing high LGR5.

## INTRODUCTION

Neuroblastoma (NB) is one of the commonest extracranial paediatric solid tumours, arising from neural crest progenitor cells of the sympathetic nervous system which have failed to undergo regulated differentiation and development as a result of aberrant gene expression programmes instigated by critical oncoproteins [1, 2]. The two best studied, and genetically modelled, drivers of NB are the transcription factor MYCN [3] and the receptor tyrosine kinase anaplastic lymphoma kinase (ALK) [4]. Gene amplification of the *MYCN* proto-oncogene occurs in about 50% of high-risk NBs (~20% of total NBs), and activating *ALK* point mutations occur in about 10% of NBs and encompass all clinical subtypes [4–6]. The oncogenic p.F1174L *ALK* mutant has been shown to potentiate the tumorigenic effect of MYCN in a mouse model, leading to higher penetrance, earlier onset and increased lethality [7]. This pronounced effect on tumorigenicity was accompanied by dramatic activation of the mitogen-activated protein kinase (MAPK) and phosphoinositide 3-kinase/Akt (PI3K/Akt) pathways. Both PI3K/Akt and MAPK signalling pathways are frequently deregulated in cancer, and represent targets for therapeutic intervention [8, 9]. Indeed elevated Akt signalling has been shown to be an indicator for poor prognosis NB [10] and PI3K inhibition can lead to decreased levels of MYCN protein in NB cells [11]. Activation of the mitogen/extracellular signal-regulated kinases (MEK1/2)/extracellular signal-regulated kinases (ERK1/2) was also frequently observed in NB [10], and a low frequency of missense mutations of genes in the Ras-Raf-MEK/ERK pathway has been reported in NB [5, 6, 12]. Importantly, two very recent studies have demonstrated that mutations in this pathway are more frequent in relapsing NB [13, 14].

Another signalling pathway that is frequently disrupted during tumorigenesis is the canonical Wnt/β-catenin pathway. Here signal transduction begins with binding of secreted Wnt ligands to Frizzled and LRP5/6 receptors, which triggers inactivation of a cytoplasmic “destruction complex” controlling the cellular pool of the transcriptional co-activator β-catenin. As β-catenin increases, it can translocate to the nucleus and activate T-cell factor/lymphoid enhancer factor (TCF/LEF) transcription factors, leading to induced expression of key genes involved in proliferation, differentiation, metabolism and stemness [15, 16]. Wnt signalling amplitude can be increased by the R-spondin family of growth factors (Rspo1-4) binding to leucine-rich repeat-containing G-protein coupled receptors (LGRs) such as LGR5 [17, 18]. The LGR-Rspo complex then recruits and promotes membrane clearance of the E3 ubiquitin ligase ZNRF3/RNF43, which would otherwise participate in turnover of the Wnt receptor complex at the cell-surface. Thus LGR-Rspo binding leads to accumulation of Frizzled and LRP6 receptors at the plasma membrane and enhanced Wnt signaling [19]. LGR5 is an established marker of the intestinal stem cell niche, and also marks stem cells in other tissues [15]. LGR5 expressing cells behave like cancer stem cells (CSCs) in breast [20] and colorectal cancer [21], glioblastoma [22], and a mouse-model of papillomavirus-induced squamous cell carcinoma [23].

Unlike many other cancers, defects in Wnt pathway genes, such as activating mutations of *CTNNB1*, encoding β-catenin, have not been found in NB, and NB cell-lines do not exhibit Wnt signalling activity measured by TCF/LEF reporter plasmids. However, tumour samples from high-risk NB patients with and without MYCN amplification express canonical Wnt pathway target genes at high levels, indicative of Wnt deregulation in NB [24]. Intriguingly, LGR5 mRNA was also shown to be elevated when NB xenografts were propagated as sphere cultures. These cultures also exhibited high expression of Wnt target genes, and displayed higher tumorigenicity *in vivo* [25].

In this study, we examine the expression and functions of LGR5 in NB. Our data suggests that LGR5 plays a key role in not only regulating Wnt signalling, but also MEK/ERK signalling in neuroblastoma cells, thereby regulating proliferation and survival respectively.

## RESULTS

### Expression pattern of LGR5 in NB cell lines and tumour tissues

**Figure 1:**
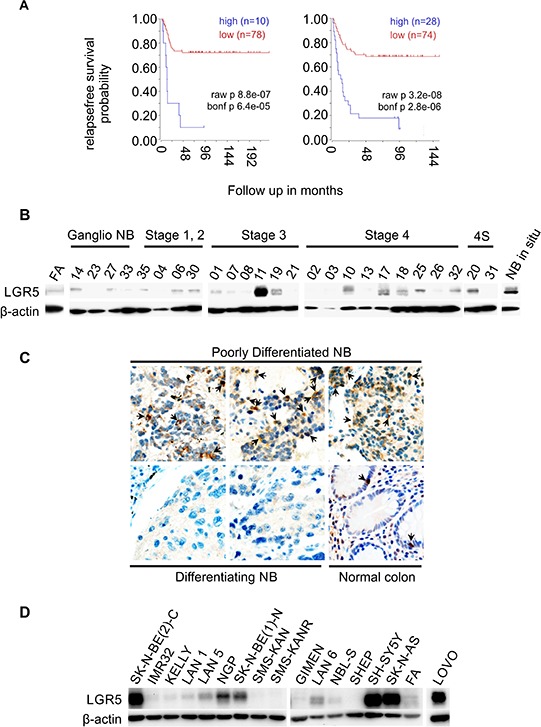
Expression of LGR5 in neuroblastoma **A.** Kaplan-Meier survival curves derived from 2 independent microarray analyses of neuroblastomas showing the association of high LGR5 mRNA expression with poor prognosis. Datasets used are from reference [27] (left) and [26] (right), obtained using the R2 Genomics Analysis and Visualization Platform (http://r2.amc.nl). **B.** Immunoblotting showing elevated expression of LGR5 in primary NBs. Fetal Adrenal protein is shown as a normal control(FA). **C.** Immunohistochemistry demonstrating prominent cytoplasmic staining in poorly differentiated NBs (top) and little or no staining in differentiating NBs (bottom). A section of normal colon where interspersed LGR5 positive cells are located at the bottom of crypts is shown as a control. **D.** Immunoblotting showing elevated expression of LGR5 in cell-lines. The colorectal cancer cell line LOVO is shown for comparison.

*In silico* analysis of NB microarray datasets was carried out using the R2 Genomics Analysis and visualization Platform (http://r2.amc.nl) in datasets with clinical correlates for both *MYCN*-unamplified and *MYCN*-amplified NBs [26, 27]. High *LGR5* mRNA expression correlated with low probability of relapse-free survival in both datasets (Bonferroni corrected *p* = 6.4^e-05^ and *p* = 2.8^e-06^, see Figure 1A). We therefore examined LGR5 protein levels in primary NBs using immunoblotting in panel of 26 NBs, and observed higher expression of LGR5 in stage 3 and 4 tumours relative to stage 1 and 2 tumours and normal fetal adrenal (Figure 1B). Immunohistochemical analysis showed that cytoplasmic LGR5 staining was observed in 9/25 poorly differentiated tumours (36%) and only 1/7 differentiating NBs (14%) (Figure 1C). Similarly 7/15 NB cell-lines showed high levels of LGR5 protein (Figure 1D). Cells with high expression did not segregate with established NB etiological factors such as *MYCN* amplification or *ALK* mutation (Supplementary Table 1). The increased expression of LGR5 in poorly differentiated and high stage primary NBs and NB cell lines suggests that certain subsets of NBs are competent for Wnt signalling dependent on Wnt and R-Spondin ligands.

### Canonical Wnt signalling capacity of LGR5 expressing NB cell lines

**Figure 2:**
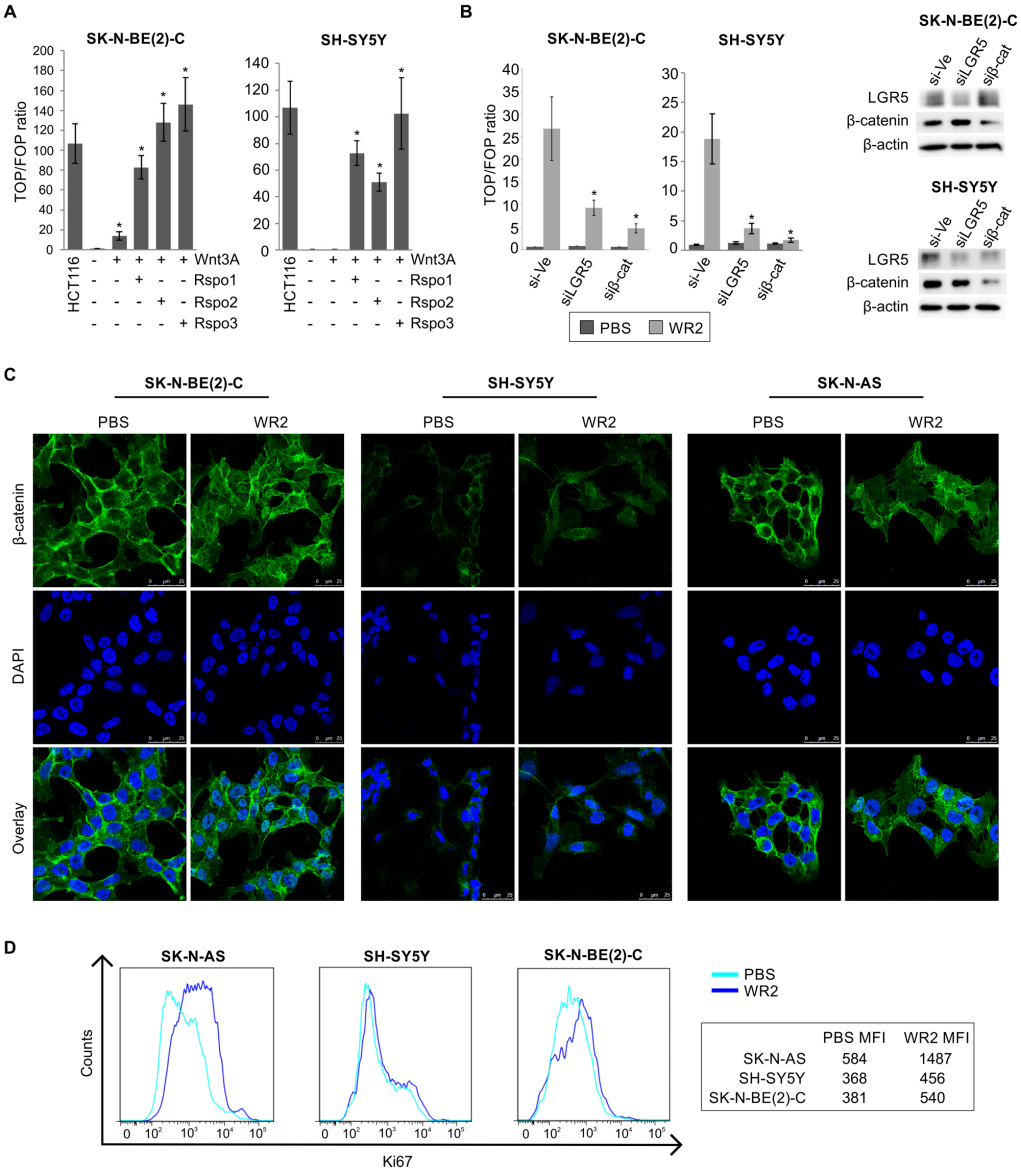
Wnt3a and R-spondin responsiveness of neuroblastoma cell lines **A.** TOPFLASH reporter assay for SK-N-BE(2)-C and SH-SY5Y showing increased luciferase activity upon co-stimulation with Wnt3a and Rspos 1–3 ( *p* < 5.0^e-04^ and *p* < 5.0^e-03^ respectively). **B.** TOPFLASH reporter assay combined with control siRNA, LGR5 or β-catenin targeting siRNAs, demonstrating requirement for LGR5 for reporter activity (* p* < 0.05). Reporter assays were conducted at least twice in triplicate. PBS: Phosphate-buffered saline, WR2: Wnt3a/Rspo2 treatment. **C.** Confocal microscopy of β-catenin nuclear translocation in SK-N-BE(2)-C, SH-SY5Y and SK-N-AS cells on addition of Wnt3a/Rspo2. Green fluorescent staining shows β-catenin and cells were counterstained with DAPI (blue) for nuclei control **D.** Flow cytometry analysis of NB lines treated with Wnt3a/Rspo2 and labelled with Ki67 to measure proliferation. Note the shift and increased median fluorescence intensity (MFI) accompanying treatment. Note that x-axes are in log scale.

The effect of Wnt3a and R-spondin ligands on stimulation of Wnt/β-catenin signalling pathway was examined by the TOPFLASH reporter assay in SK-N-BE(2)-C and SH-SY5Y cell lines, which represent *MYCN*-amplified and *ALK*-mutated cells respectively (see Supplementary Table 1). Wnt3a treatment alone increased TOPFLASH activity in SK-N-BE(2)-C by approximately 14-fold, but addition of any of the R-spondins 1–3 dramatically increased TOPFLASH activity to 83-fold with Rspo1, 129-fold with Rspo2 and 147-fold with Rspo3 (*p* < 5.0^e-04^). In the case of SH-SY5Y cells, no TOPFLASH induction was apparent with Wnt3a alone, but a 50–100 fold increase was apparent on addition of Rspo ligands (*p* < 5.0^e-03^). The Wnt activity in the Wnt3a/Rspo-treated SK-N-BE(2)-C and SH-SY5Y cells was comparable to the colorectal cancer line HCT116 which has an activating point-mutation of β-catenin and constitutive Wnt activation (Figure 2A). Similar Wnt/Rspo2 responsiveness was also observed in the SK-N-AS cell-line (Supplementary Figure 1). To confirm that TOPFLASH activity was indeed reliant on LGR5, we first knocked down LGR5 in SK-N-BE(2)-C and SH-SY5Y cells, and then treated them with Wnt3a/Rspo2 before reporter assays. As shown in Figure 2B, knockdown of either LGR5 or β-catenin led to greatly reduced TOPFLASH activity.

We next evaluated whether co-stimulation with Wnt3a/Rspo2 resulted in nuclear translocation of β-catenin using confocal microscopy. Distinct nuclear translocation of β-catenin was visible in SK-N-BE(2)-C, SH-SY5Y and SK-N-AS cell lines when stimulated with Wnt3a/Rspo2 (Figure 2C). To evaluate the effect of Wnt3a/Rspo2 on cell growth, we monitored the median fluorescence intensity (MFI) of the proliferation marker Ki67 using flow cytometry, and found increases accompanying Wnt3a/Rspo2 treatment in all three lines tested, especially SK-N-AS and SK-N-BE(2)-C (Figure 2D).

In order to further confirm Wnt activation, we analysed induction of several established target genes of canonical Wnt signalling in the NB cell lines treated with Wnt3a/Rspo2, including *AXIN2*, *BMP4*, *JAG1*, *TNFRSF19*, and *TWIST1* (http://web.stanford.edu/group/nusselab/cgi-bin/wnt/). Robust induction of all these genes was observed (Supplementary Figure 2A). We also evaluated *MYC* and *MYCN*, which are also reported Wnt targets, but neither was markedly elevated. c-MYC and MYCN protein expression was also examined, and showed that neither c-MYC (SK-N-AS and SH-SY5Y) or MYCN (SK-N-BE(2)-C) was upregulated with Wnt3a/Rspo2 treatment (Supplementary Figure 2B).

Taken together these studies establish LGR5 as a key mediator of proliferative canonical Wnt signalling in neuroblastoma cells.

### LGR5 depletion induces apoptosis of NB cells independent of Wnt/β-catenin signalling

**Figure 3:**
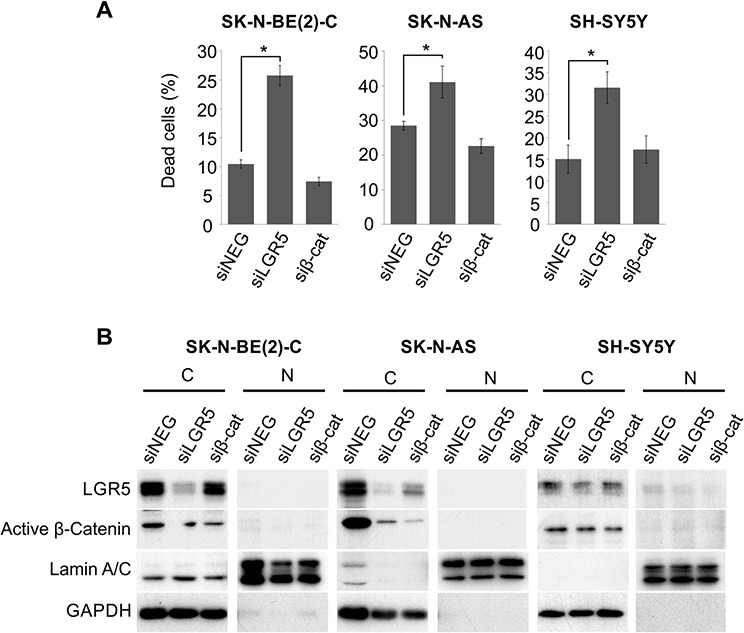
LGR5 knockdown-induced cell death is independent of Wnt/β-catenin signalling pathway **A.** SK-N-BE(2)-C, SK-N-AS and SH-SY5Y cells were transfected with siRNAs targeting LGR5 or β-catenin. LGR5 knockdown led to dramatic cell death in all three cell lines tested ( *p* < 0.05), but β-catenin depletion did not. **B.** Immunoblots confirming knockdowns and the absence of dephosphorylated (active) β-catenin. C: cytoplasmic, N: nuclear.

During the course of our TOPFLASH experiments, we observed that knockdown of LGR5 for greater than 48 hrs had a dramatic effect on cell survival. We therefore sought to further characterise the effects of LGR5 depletion in our panel of NB lines. Although there is no constitutive Wnt signalling apparent in NB cell lines as assessed by the TOPFLASH reporter assay (Figure 2, [24]), we further assessed whether the cell-death induced by LGR5 depletion involved a low residual level of Wnt/β-catenin signalling. Whereas LGR5 knockdowns in all three lines induced significant cell-death (*p* < 0.05), no effects were observed in β-catenin knockdowns (Figure 3). Transcriptionally active β-catenin is unphosphorylated at critical serine and threonine residues, and can be specifically detected with an anti-active β-catenin antibody [28]. Our immunoblotting demonstrates that active β-catenin was not detected in the nuclear fractions from the 3 cell lines (Figure 3B).

**Figure 4:**
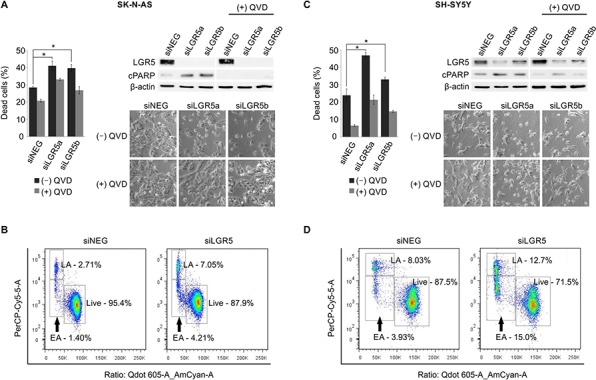
Depletion of LGR5 induces apoptosis in neuroblastoma cells Knockdown of LGR5 with independent siRNAs induces apoptosis shown by cell-counts, increased cleaved PARP (cPARP), and rescue by the caspase 3 inhibitor QVD **A.** SK-N-AS cells and **B.** SH-SY5Y cells. Asterisks denote *p* < 0.05, and assays are representative of at least 3 biological replicates. Membrane asymmetry assessment of apoptosis in **C.** SK-N-AS cells and **D.** SH-SY5Y cells. EA, early apoptosis; LA, late apoptosis.

In both SK-N-AS and SH-SY5Y cells, knockdown of LGR5 with 2 independent siRNAs was accompanied by visible cell death (*p* < 0.05). Immunoblotting showed an increase in cleaved poly ADP ribose polymerase (PARP) in LGR5 knockdowns, and cell death was rescued by treatment with the caspase inhibitor Q-VD-OPh (QVD). Flow cytometry measuring membrane asymmetry also confirmed apoptotic cell death (Figure 4). Similar apoptosis accompanying LGR5 depletion was observed in SK-N-BE(2)-C cells (Supplementary Figure 3).

Together with our reporter assays, these experiments confirm that there is no basal Wnt signalling in the NB cell lines examined, and that the dramatic effects of LGR5 knockdown must be attributable to other signalling pathways.

### LGR5 regulates MEK/ERK and Akt signalling pathways

**Figure 5:**
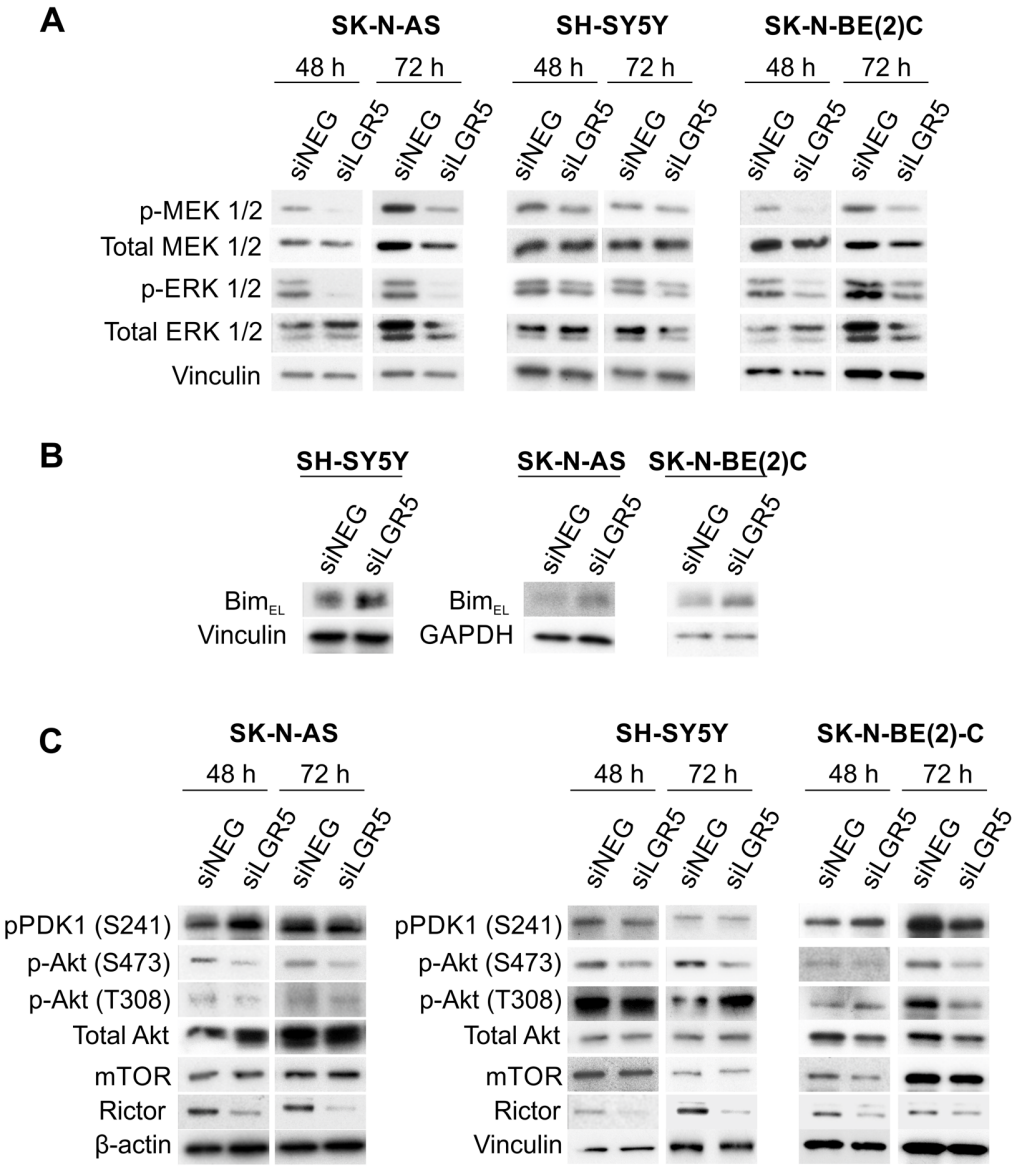
LGR5 regulates MEK/ERK and Akt signalling Immunoblotting demonstrating **A.** decreases in p-ERK1/2 (T202/Y204) and p-MEK1/2 (S217/221), **B.** Elevated Bim-EL and **C.** altered Akt phosphorylation and Rictor levels accompanying LGR5 knockdown.

As cancer cell survival can involve MEK/ERK and Akt signalling pathways [9, 29], we assessed whether LGR5 knockdown had any effects on the activated (phosphorylated) forms of these kinases. Both phospho-serine MEK1/2 (S217/221) and phospho-serine ERK1/2 (T202/Y204) were dramatically decreased in SK-N-AS and SK-N-BE(2)-C cell lines, with SH-SY5Y cells also displaying a decrease, albeit slightly less pronounced. Total MEK and ERK were not changed after 48 hrs of knockdown, but showed a slight decrease after 72 hrs (Figure 5A). We also assessed total c-Raf and phospho-serine (S338) c-Raf as Raf is an intermediary of Ras-MEK-ERK signalling. However, no changes in total or phospho-c-RAF(S338) were apparent (data not shown). As the BH3-only protein Bim (Bcl-2 interacting mediator of cell death) is directly phosphorylated by ERK1/2, leading to its degradation and unavailability to pro-survival members of the Bcl-2 family [30, 31], we assessed the effect of LGR5 depletion on Bim_EL_ by immunoblotting. Knockdowns in all 3 cell-lines tested resulted in elevated Bim_EL_, supporting LGR5-ERK-Bim signalling as a pro-survival pathway in NB (Figure 5B).

We also examined phosphorylated (S473 and T308) and total Akt changes. Whilst the PDK1-mediated phosphorylation of T308 remained unchanged, phosphorylation of S473 was greatly diminished following LGR5 knockdown (Figure 5C). Consistent with the unaltered p-Akt(T308), phospho-PDK1 (S241) was also unaffected. As PDK1 is downstream of phosphatidylinositide 3-kinase (PI3K), this data suggests that early LGR5 effects are not linked to the PI3K pathway, although at 72 hrs some decreases are apparent. Early effects on Akt signalling largely affect p-Akt(S473) in all cell lines. This modification is known to depend on by the Rictor-mTOR complex [32], and consistent with this, decreased Rictor is apparent following LGR5 knockdown in all cell-lines, whereas mTOR is unaltered except in SK-N-BE(2)-C cells.

The SH-SY5Y cell line is known to have an activating mutation of the *ALK* gene [4], and mutant ALK activates MEK/ERK signalling [7]. We therefore examined whether LGR5 knockdown might lead to a reduction in ALK and phospho-ALK levels. However, in both SH-SY5Y and SK-N-BE(2)-C lines, phospho-ALK was unchanged and a slight increase of total ALK was observed in SH-SY5Y cells (Supplementary Figure 4). Thus LGR5 does not exert its effect on MEK/ERK by influencing ALK activity. As the data above indicate different modes of signalling via LGR5, we tested whether the Wnt3a/Rspo2 induced proliferation (Figure 2D) also necessitated increased MEK/ERK signalling. In contrast to the positive control for induction of proliferation, epidermal growth factor (EGF), Wnt3a/Rspo2 treatment did not lead to increased pMEK1/2 and pERK1/2 (Supplementary Figure 5).

Together these experiments demonstrate previously unknown pro-survival signalling activities mediated by LGR5 in addition to its well-established role in Wnt signalling amplification.

### LGR5 is required for the G1 to S-phase cell cycle transition

**Figure 6:**
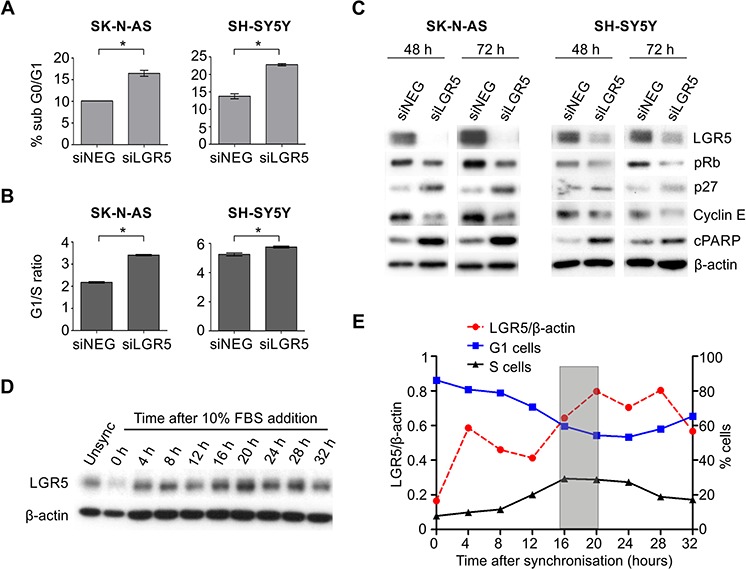
LGR5 is required for G1 to S phase transition **A.** Increased sub-G0/G1 cells following LGR5 knockdown of SK-N-AS and SH-SY5Y cells demonstrated by flow cytometry. Asterisks denote *p* < 0.05. **B.** Cell cycle analysis of LGR5 knockdowns. After 48 hrs of transfection, G1 arrest of cells is apparent through comparison of G1-S phase ratios. Asterisks denote *p* < 0.05. **C.** Immunoblotting analysis of cell cycle regulatory proteins confirming G1 arrest accompanying LGR5 knockdown.**D.** Immunoblot demonstrating cell cycle dependent changes in LGR5 expression in SH-SY5Y cells. **E.** Quantification of LGR5 protein levels (relative to β-actin) plotted against percentage of cells in G1- or S-phase.

The dramatic effects of LGR5 levels on MEK/ERK signalling suggested to us that LGR5 may be involved in cell cycle regulation, as the MEK/ERK pathway is known to be a master regulator of the G1- to S-phase transition [33]. Depletion of LGR5 in SK-N-AS and SH-SY5Y cells led to a significant increase in the sub-G0/G1 population after a 72 hrs knockdown (Figure 6A). Evaluating cell cycle changes earlier (48 hrs) showed a significant increase in the G1/S-phase ratio, and that the cells accumulated in G1 phase, indicative of G1 arrest (Figure 6B). Further, immunoblotting analysis of proteins involved in cell cycle progression showed a down regulation in the expression of phosphorylated Rb and cyclin E along with an increase in the expression of cell cycle inhibitor p27 (Figure 6C), thereby confirming G1 arrest of cells following LGR5 depletion. As these results indicated that LGR5 is required for G1 to S phase transition of cell cycle, we also investigated the expression of LGR5 during the cell cycle. SH-SY5Y cells were synchronised by serum starvation, fed with serum containing media after 48 hrs, and samples collected at 4 hr intervals. Immunoblotting demonstrated low LGR5 protein at 0 hrs when >85% of cells were in G1 phase, with increasing LGR5 as cells underwent a G1-S phase transition (16–20 hrs, Figure 6D). A strong inverse correlation between the percentage of cells distributed in G1 phase and expression of LGR5 is apparent (Supplementary Figure 6).

These data demonstrate for the first time that LGR5 has a role in regulating the cell cycle, and further emphasise the importance of LGR5 in regulating NB cell growth.

## DISCUSSION

**Figure 7:**
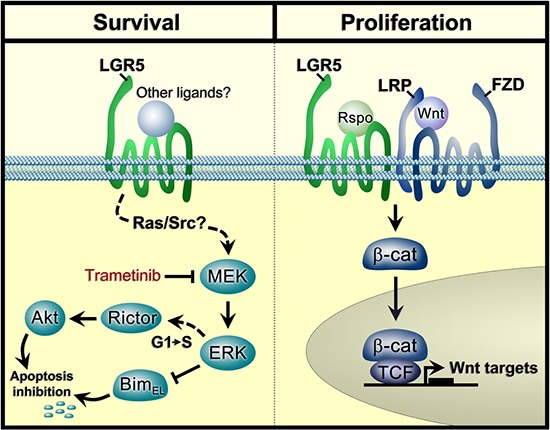
A model showing regulatory modalities for LGR5 in neuroblastoma Pro-survival signalling of MEK/ERK activities which are R-Spondin independent are shown on the left, and may be inhibited by MEK/ERK inhibitors such as Trametinib. Proliferative Wnt signalling in the presence of Wnt/Rspos is shown on the right. FZD: Frizzled receptors; LRP: Low Density Lipoprotein Receptor-Related Proteins, β-cat: β-catenin, TCF: T-cell factor/lymphoid enhancer factor.

LGR5 has been shown to be highly expressed in a number of cancers, including colorectal cancer [34], breast cancer [20], cervical cancer [35] and glioblastoma [36], where it is associated with positive modulation of Wnt signalling in cancer stem cells. In this study, we demonstrate for the first time that LGR5 is also highly expressed in NB, where it modulates Wnt signalling associated with increased proliferation. Importantly, we also demonstrate novel regulatory properties of LGR5 upstream of MEK/ERK and Akt pro-survival signalling, pathways that are frequently activated in primary neuroblastomas [10] (Figure 7).

Previous data has shown that NB cell lines do not exhibit any basal β-catenin-dependent TCF activity [24]. Consistent with this, we did not observe any reporter activity with neuroblastoma cell lines in the absence of Wnt3a and R-spondin ligands. Contrary to a previous report [33], however, we did not detect aberrant nuclear β-catenin in either *MYCN*-unamplified or *MYCN*-amplified NB lines, presumably because we used an antibody that specifically detects the active unphosphorylated form of β-catenin rather than total β-catenin [28]. Whilst this and another previous study have associated increased Wnt signalling with increased tumorigenicity based on induction of Wnt target genes in primary tumours and the development of highly tumorigenic sphere cultures [24, 25], our studies further directly demonstrate the proliferative capacity of Wnt3a/Rspo2 on NB cells expressing LGR5. Wnt inducibility does not clearly segregate with any particular genotype, although we note that SH-SY5Y showed the smallest response to Wnt3a/Rspo2, probably due to ALK being its major driver. Our studies did not support the notion that aberrant Wnt signalling explains high c-MYC expression in *MYCN*-unamplified NB lines as previously suggested [24], as Wnt3a/Rspo2 treatment of SK-N-AS and SH-SY5Y cells contrarily results in a slight decrease of c-MYC. A similar effect is also observed for MYCN in SK-N-BE(2)-C cells, and a significant transcriptional induction of *MYC* or *MYCN* genes after Wnt/Rspo2 treatment of SK-N-BE(2)-C cells was not observed (Supplementary Figure 2). One possible explanation for this in SK-N-AS cells is the reduced MEK/ERK observed upon Wnt3a/Rspo2 stimulation (Supplementary Figure 5), as phosphorylation by this MAPK pathway has been shown to be involved in the stabilising phosphorylation of Serine 62 of c-MYC [37]. Context-dependent crosstalk between the Wnt and Ras-Raf-MEK/ERK signalling has been shown previously, with Wnt signalling stabilising Ras in intestinal cancers [38], and the MAPK pathway repressing Wnt signalling in melanoma [39]. Whilst this is not the focus of our study and our data is limited to one time-point, it is possible that Wnt signalling in SK-N-AS cells dampens MEK/ERK signalling. Further transcriptomic and proteomic analyses of Wnt induction of NB in the future will help illuminate other growth regulatory and cross-talk mechanisms that may be involved in Wnt/Rspo2 induced proliferation. In SH-SY5Y or SK-N-BE(2)-C cells, decreased c-MYC and MYCN may rather be associated with lower Akt signalling, inhibition of PI3K/Akt having previously been shown to reduce MYCN levels [11].

Intriguingly, LGR5 knockdown resulted in pronounced cell death through apoptosis that was not apparent when β-catenin was knocked down. This demonstrated that LGR5 depletion mediated cell death is independent of Wnt/β-catenin signalling. Thus in addition to playing an important role in Wnt stimulated proliferation of NB cells, LGR5 is also required for the survival of NB cells. LGR5 knockdown in all three of our tested cell lines led to a dramatic reduction in phosphorylated MEK/ERK which is a critical mediator of tumour cell survival [40] and repressor of apoptosis [30, 31]. MEK/ERK diminution was accompanied by an increase of the pro-apoptotic tumour suppressor Bim_EL_. This capacity of LGR5 is reminiscent of brain-derived neurotrophic factor (BDNF)/tyrosine kinase receptor (TrkB) axis which has been shown to repress Bim via MEK/ERK phosphorylation [41] leading to increased resistance to chemotherapy in NB [42]. It will therefore be of great interest to examine LGR5 expression in a larger cohort of patients using immunohistochemistry to evaluate its prognostic significance, and assess its correlation with phosphorylated MEK/ERK/Akt in primary tumours. The ability of LGR5, a marker of stem cells in a variety of tissues [43], to govern MEK/ERK activity may also be integral to its role in stem cell maintenance, as high levels of MEK/ERK have been shown to be required for maintaining human embryonic stem cells in an undifferentiated state [44]. We did not detect upstream changes in total or phosphorylated (S338) c-Raf at time-points where phosphorylated MEK/ERK was altered, suggesting that LGR5-mediated regulation of MEK/ERK in NB cells is Raf-independent. Cells expressing ALK proteins have been shown to mediate MEK/ERK signalling independent of c-Raf, an effect that was attributable to an as yet unidentified kinase [45]. However, Raf phosphorylation patterns are complex and it remains possible that alternative post-translational modifications of Raf are involved [46]. Other kinases capable of Raf-independent MEK/ERK activation such as COT/MAP3K8 [47] may also be involved.

Two of our three cell lines have *NRAS* mutations (SK-N-AS [12] and SH-SY5Y [48]), and, while the SK-N-BE(2)-C line does not (our unpublished data and reference 16), it has inactivating mutations of *NF1*, providing an alternate mechanism for activation of Ras signalling via neurofibromin down-regulation [49]. However while known to have mutant *NRAS*, SH-SY5Y cells reportedly display no activated Ras-GTP [50]. Together this evidence suggests that the survival interference triggered by LGR5 depletion may involve not only Ras, but also other signal transduction pathways, and it is pertinent to note that GPCRs have been shown to signal directly to Src kinases [51] which also contribute to MEK/ERK activation [52]. Whilst a low level of Ras-Raf-MEK/ERK pathways mutations have previously been observed in NB [5, 6], two very recent studies strongly associate Ras/MAPK pathway mutations with relapsing NB [13, 14]. Intriguingly, both these studies each include an individual relapsed tumour with missense mutations of *LGR5*, ie 2 mutations in a total of 39 relapsed tumours analysed (5%). Our functional analyses suggest that LGR5 might also be included in the mutational geneset of the Ras/MAPK pathway, and further strengthens the importance of Ras/MAPK to NB progression. Consistent with the notion of an LGR5-Ras-MEK/ERK axis, we have observed that treatment of our NB cell lines with Trametinib/GSK1120212 [53], a potent inhibitor of MEK1/2, leads to a rapid elimination of LGR5 (data not shown).

ERK1/2 signalling is known to play a crucial role in the G1-S phase progression of the cell cycle by negatively regulating the cell cycle inhibitor p27 [33], and consistent with this, reduction of MEK/ERK signalling in LGR5 depleted neuroblastoma cells led to a G1-phase cell cycle arrest. We also demonstrated that LGR5 is cell cycle regulated, being low in early G1 phase, peaking at G1-S phase transition, and then decreasing as cells progressed through S phase. These observations may have important implications for studies exploring LGR5 regulation and those employing LGR5 as a marker of cell populations. Agents inducing G1-phase cell cycle arrest, for example, will appear to downregulate LGR5, although this effect may be indirect and attributable to the cell cycle distribution of the analysed cells. Similarly, cell-sorting of side-populations contingent on high LGR5 expression levels may be obfuscated by fluctuating expression levels during the cell cycle.

Our data also suggested that LGR5 may be upstream of Akt signalling. However, the pattern of Akt and PDK1 modifications suggests that rather than a direct effect (via PI3K/PDK1), or a kinase-dependent ERK/Akt crosstalk [54], the effects observed are due to the cell cycle arrest induced by LGR5 knockdown. The fact that only p-Akt (S473) decreased, whereas p-Akt (T308) did not, implicated mTORC2 involvement. This was further narrowed down to Rictor, which decreased dramatically, in contrast to mTOR which was unchanged. It was recently demonstrated that Rictor mRNA translation is exquisitely regulated at the G1-S transition, rising sharply in S-phase and phosphorylating p-Akt (S473) [55]. We therefore interpret the LGR5 depletion-induced effects on Akt as indirect and attributable to cell cycle arrest.

In summary, our data presented here highlights LGR5 as a candidate oncoprotein mediating key signalling pathways in NB and possibly other cancers, such as breast and cervical cancer, and glioblastoma. Antibodies such as BNC101 which target LGR5 may therefore a powerful therapeutic agents to be deployed in combination with kinase inhibitor therapies, particularly those targeting the MEK/ERK pathway such as Trametinib [53].

## MATERIALS AND METHODS

### Patient samples

All tissues were obtained as snap frozen samples from the Bristol Children's Hospital. Details of clinical samples are given in Supplementary Table 2. For immunohistochemistry, neuroblastomas were from archival tissues collected at The Children's Hospital at Westmead Histopathology Department (Sydney, Australia) since 1950, and assembled into a tissue microarray. All human tissues were acquired with appropriate local research ethics committee approval.

### Tissue culture

The NB cell lines used in this study are SK-N-BE(2)-C, IMR32, NGP (kindly provided by Pramila Ramani, Bristol Royal Infirmary, University of Bristol), GIMEN, SH-5Y 5Y (kindly provided by Carmel McConville, University of Birmingham), SK-N-BE(1)N, SHEP (kindly provided by Robert A Ross, Fordham University), SMS-KAN, SMS-KANR, LAN5, LAN6 (purchased from the Children`s Oncology Group, USA), LAN1, KELLY, NBL-S (purchased from the Leibniz-Institut DSMZ - Deutsche Sammlung von Mikroorganismen und Zellkulturen GmbH) and SK-N-AS (Sigma). Cells were cultured in Dulbecco's modified Eagle's medium (DMEM) mixture F12-HAM (Sigma) supplemented with 10% fetal bovine serum (FBS) (PAA cell culture), 200 mM L-Glutamine (Sigma), 100 mM penicillin, 0.1 mg/mL streptomycin (Sigma) and 1% v/v non-essential amino acids (Life technologies) as adherent monolayers and maintained in a humidified incubator at 37°C with 5% CO2.

### siRNA knockdown in neuroblastoma cell lines

Knockdowns and cell treatments with QVD (quinolyl-valyl-O-methylaspartyl-(-2,6-difluorophenoxy)-methyl ketone, Sigma) were carried out with Lipofectamine 2000 (Invitrogen) essentially as previously described [56]. Cells were transfected with 50 nM of small interfering RNA (siRNA) targeting LGR5. Individual siRNAs from the Dharmacon Smartpool of 4 siRNAs were used for LGR5 (DharmaconGE), as well as a fifth siRNA (5′-GCAUUUGUAGGCAACCCUU-3′). For *CTNNB1*, the siRNA was 5′-GGACCUAUACUUACGAAA-3′ (Sigma). For QVD treatment at 10 μM cells were incubated for 24 hrs before addition of the drug. Cells were then incubated for a further 48 hrs before harvesting, staining with Trypan Blue, and counting with an automated cell counter (Invitrogen) for the number of live and dead cells.

### Protein expression analysis

Cells were lysed with cold cell lysis buffer (Cell Signalling Technology) with protease inhibitor (Roche) and phosphatase inhibitor (Roche). For preparation of cytoplasmic and nuclear protein fractions, cells were lysed as described previously, left on ice for 15 minutes and spun for 10 minutes at 13000rpm. The supernatant (cytoplasmic fraction) was transferred to a new tube and the remaining pellet was washed with PBS and lysed with 1x nuclear lysis buffer (50 mM Tris-HCL (pH 8.1); 5 mM EDTA; 1% SDS) (nuclear fraction). The determination of cellular protein was done in accordance to a series of BSA standards using the Protein Assay kit (Bio-Rad) based on the Lowry assay [57]. Proteins were run in 10–15% acrylamide gel (Severn Biotech) then transferred to a Immobilon-P PVDF membrane (Millipore) for probing with primary antibodies (listed in Supplementary Table 3).

Immunohistochemistry was performed with a Leica Microsystem Bond III automated machine using the Bond Polymer Refine Detection Kit (Ref DS9800) followed by Bond Dab Enhancer (AR9432). The slides were dewaxed with Bond Dewax Solution (AR9222). Heat mediated antigen retrieval was performed using Bond Epitope Retrieval Solution for 10 mins. Primary antibody dilution for anti-LGR5 (Proteintech 21833-1-AP) was1:400.

### Wnt/β-catenin (TCF/LEF) reporter assays

TOPFLASH reporter assays were carried out as previously described with Super 8xTOPFLASH/FOPFLASH (kindly supplied by R. Moon) [58]. Cells were seeded at a density of 5 × 10^4^cells per well and incubated for 24 h before serum starvation and transfection with 8xTOPFLASH/FOPFLASH at 100ng/ mL then incubated for a further 24 h. Cells were treated with recombinant human Wnt3a and/or R-spondins (Rspos) 1, 2 and 3 (R&D Systems) at 50ng/mL and the activity was measured after 24 hrs using a luminometer (Modulus single tube multimode reader, Biosystems). For transfection with *LGR5* or *CTNNB1* siRNA (50 nM), Dharmafect Duo (Thermo Scientific) was used. Twenty four hrs after transfection the cells were treated with Wnt3a and R-spondin 2 at 50 ng/mL each and the activity measured 24 hrs after treatment. Phosphate Buffered Saline (PBS)/0.02% (w/v) BSA was used as a control.

### Confocal immunofluorescence

Cells were seeded onto Poly-L-lysine (Sigma) coated coverslips at a concentration of 3 × 10^5^ cells/ well. Cells were serum starved for 24 hrs before treatment with Wnt3a and Rspo2 at 50 ng/mL and PBS/0.2% (w/v) BSA was used as a control. SK-N-BE(2)-C, SH-SY5Y and SK-N-AS were fixed in 2% (v/v) paraformaldehyde (Sigma), permeabilised with 0.1% (v/v) Triton X-100 and stained with IgG1a monoclonal anti-β-catenin antibody (BD Biosciences). The threshold for β-catenin fluorescence was determined using isotype-matched primary control antibody IgG1 k Isotype (BD Biosciences). Throughout image acquisition only whole nucleated cells were captured as determined by DAPI (4, 6 Diamino-2-Phenylindole, D9542, Sigma) staining. Confocal immunofluorescence was achieved using the resonant scanning head of a Leica TCS SP2 confocal laser microscope with a 63x oil immersion objective NA 1.32 (HCX-PL-APO) assisted by Leica™ Confocal Software (LCS) version 2.61 (Leica Microsystems, Heidelberg, Germany).

### Ki67 proliferation assay

Cells were seeded at a density of 10^6^ cells/well and serum starved for 24 hrs before treatment with epidermal growth factor (sigma) at 100 ng/mL, Wnt3a and Rspo2 at 100 ng/mL. PBS/0.02% (w/v) BSA was used as a control. After 24 hrs cells were fixed with cold 70% ethanol and stained with PE Mouse Anti-Human Ki-67 according to the manufacturer's protocol (BD Pharmingen). For measuring the threshold for Ki67 staining PE mouse IgG, Ƙ isotype control (BD Pharmingen) was used. Fluorescence of Ki67 stained cells was measured using a LSRFortessa X20 (BD Biosciences) using FACS DIVA8 software (Becton-Dickinson Immunocytometry Systems, San Jose, CA, USA) and at least 20,000 events were collected. The results were analysed using FlowJo VX.0.7 analysis software (Tree Star, Inc.).

### Cell cycle analysis

Cells were seeded at a density of 10^6^ cells/well and transfected with siRNA targeting *LGR5*. After 48–72 hrs of incubation cells were washed with PBS and fixed with cold 70% ethanol overnight before treatment with RNase A (Qiagen) at 100 μg/mL and stained with 50 μg/mL of Propidium Iodide (PI) (Sigma) in the dark for 30 minutes at 37°C. DNA content of PI stained cells was measured as above.

For cell cycle synchronization analysis cells were serum deprived to promote cell cycle arrest. Medium containing 10% FBS was added to release cells from the arrest and samples were collected for protein expression analysis or fixed for cell cycle analysis every 4 hrs (0–32 hrs).

### Violet ratiometric membrane asymmetry

Cells were seeded at a density of 10^6^ cells/well and transfected with siRNA targeting *LGR5.* 72 hrs after transfection cells were washed with PBS and stained with F2N12S at 200nM and SYTOX^®^ AADvanced™ dead cell stain (Life Technologies) at 1 nM and incubated for 5 mins in the dark at room temperature. Membrane asymmetry detection was measured in a BD LSR II flow cytometer (Becton-Dickinson Immunocytometry Systems, San Jose, CA, USA) using FACS DIVA8 software (Becton-Dickinson Immunocytometry Systems, San Jose, CA, USA). At least 20,000 events were collected and the data was analysed using FlowJo VX.0. 7 analysis software (Tree Star, Inc.).

### Wnt target gene transcript analysis

RNA purification and quantitative reverse-transcriptase polymerase chain reaction (qRT-PCR) was carried out as before [56]. Serum-starved SK-N-BE(2)-C cells were stimulated with Wnt and R-spondin2 for 6 hrs. Primer sequences are given in Supplementary Table 4.

## SUPPLEMENTARY MATERIALS AND METHODS, REFERENCES FIGURES AND TABLES


