# Correction: LGR5 regulates pro-survival MEK/ERK and proliferative Wnt/β-catenin signalling in neuroblastoma

**DOI:** 10.18632/oncotarget.17685

**Published:** 2017-05-08

**Authors:** Gabriella Cunha Vieira, S. Chockalingam, Zsombor Melegh, Alexander Greenhough, Sally Malik, Marianna Szemes, Ji Hyun Park, Abderrahmane Kaidi, Li Zhou, Daniel Catchpoole, Rhys Morgan, David O. Bates, Peter J. Gabb, Karim Malik

**Present**: The originally supplied Figure 5 contains duplicate total-ERK panels.

**Correct: **The proper Figure 5 appears below. The authors sincerely apologize for this error.

Original article: Oncotarget. 2015; 6:40053-67. doi: 10.18632/oncotarget.5548

**Figure 5:**
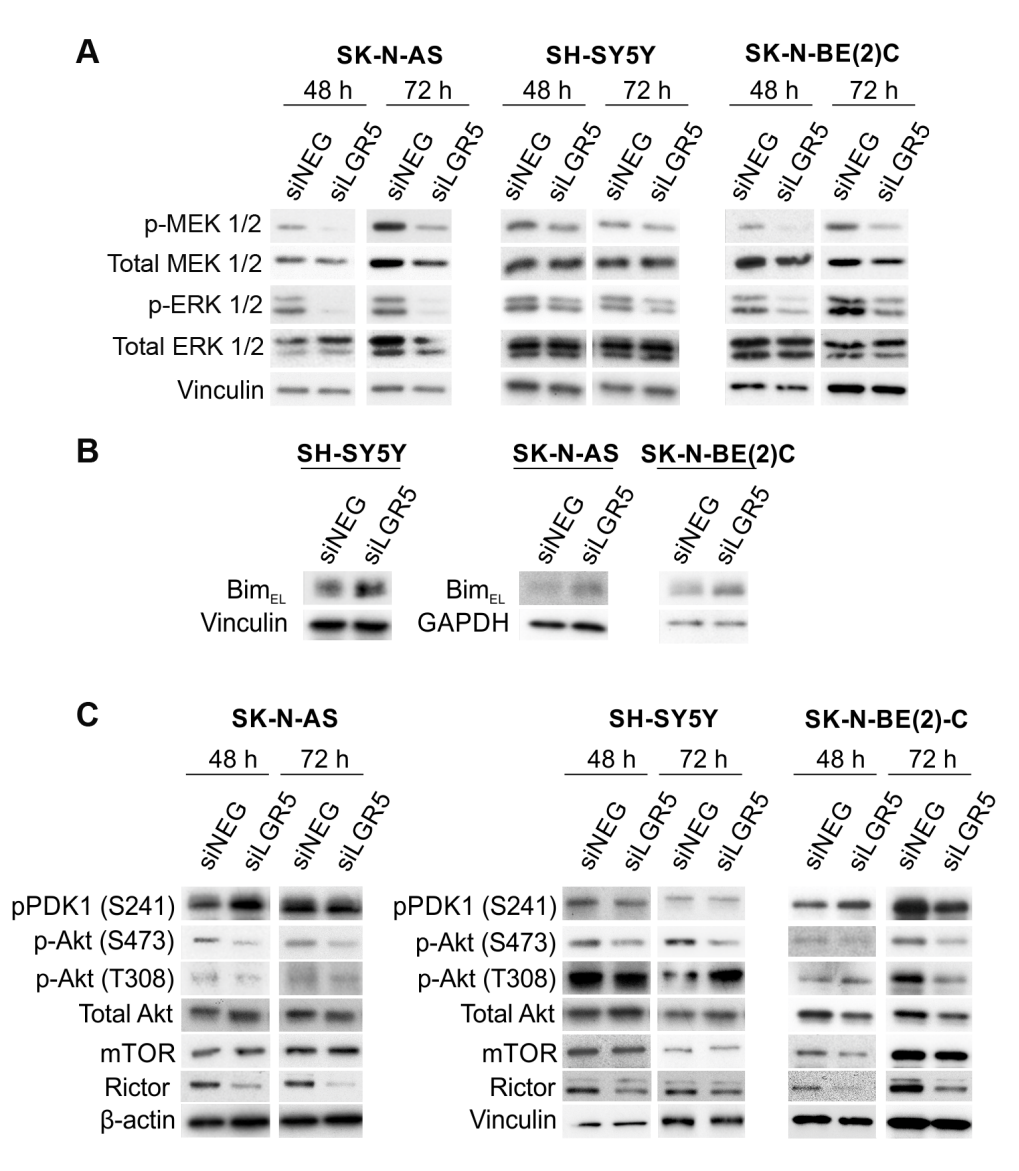
LGR5 regulates MEK/ERK and Akt signalling Immunoblotting demonstrating **A.** decreases in p-ERK1/2 (T202/Y204) and p-MEK1/2 (S217/221), **B.** Elevated Bim-EL and **C.** altered Akt phosphorylation and Rictor levels accompanying LGR5 knockdown.

