# Supplementary material for: LGR5 regulates pro-survival MEK/ERK and proliferative Wnt/β-catenin signalling in neuroblastoma
Source: Oncotarget. 2015 Oct 23;6(37):40053–67. doi: 10.18632/oncotarget.5548 (PMC4741879; doi:10.18632/oncotarget.5548)
Supplement: Supplementary file 1 [file oncotarget-06-40053-s001.pdf]

SUPPLEMENTARY MATERIALS AND METHODS

SUPPLEMENTARY REFERENCES

1. Mosse YP, Laudenslager M, Longo L, Cole KA, Wood A, Attiyeh EF, et al. Identification of ALK as a major familial neuroblastoma predisposition gene. Nature. 2008; 455:930–5.
2. George RE, Sanda T, Hanna M, Frohling S, Luther W, 2nd, Zhang J, et al. Activating mutations in ALK provide a therapeutic target in neuroblastoma. Nature. 2008; 455:975–8.
3. Chen Y, Takita J, Choi YL, Kato M, Ohira M, Sanada M, et al. Oncogenic mutations of ALK kinase in neuroblastoma. Nature. 2008;455(7215):971-4.
4. Nakamura Y, Ozaki T, Niizuma H, Ohira M, Kamijo T, Nakagawara A. Functional characterization of a new p53 mutant generated by homozygous deletion in a neuroblastoma cell line. Biochem Biophys Res Commun. 2007; 354:892–8.
5. Tweddle DA, Pearson AD, Haber M, Norris MD, Xue C, Flemming C, et al. The p53 pathway and its inactivation in neuroblastoma. Cancer Lett. 2003; 197:93–8.
6. Van Maerken T, Rihani A, Dreidax D, De Clercq S, Yigit N, Marine JC, et al. Functional analysis of the p53 pathway in neuroblastoma cells using the small-molecule MDM2 antagonist nutlin-3. Mol Cancer Ther. 2011; 10:983–93.
7. Goldschneider D, Horvilleur E, Plassa LF, Guillaud-Bataille M, Million K, Wittmer-Dupret E, et al. Expression of C-terminal deleted p53 isoforms in neuroblastoma. Nucleic acids research. 2006; 34:5603–12.
8. Holzel M, Huang S, Koster J, Ora I, Lakeman A, Caron H, et al. NF1 is a tumor suppressor in neuroblastoma that determines retinoic acid response and disease outcome. Cell. 2010; 142:218–29.
9. The I, Murthy AE, Hannigan GE, Jacoby LB, Menon AG, Gusella JF, et al. Neurofibromatosis type 1 gene mutations in neuroblastoma. Nat Genet. 1993; 3:62– 6.

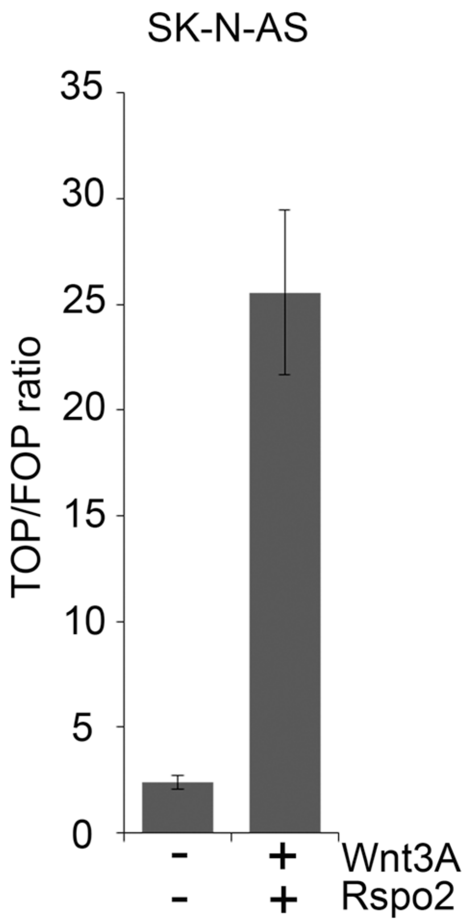

Supplementary Figure S1: Wnt3a/Rspo2 activation of TOPLASH reporter in SK-N-AS cells. Induction of TOPFLASH is shown relative to FOPFLASH activity.

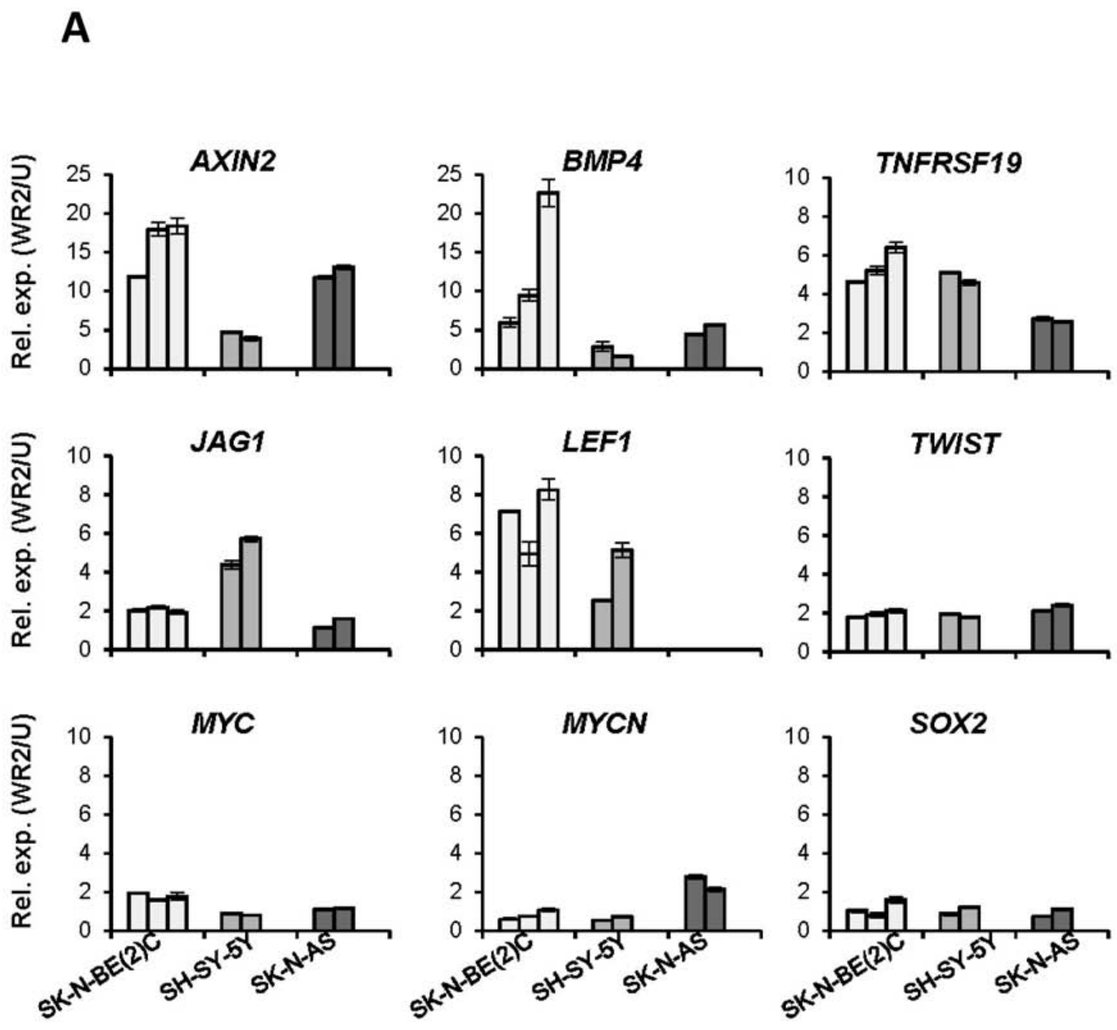

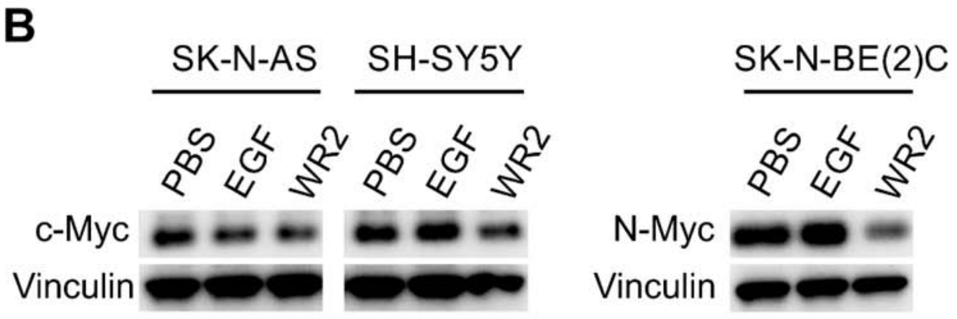

Supplementary Figure S2: Activation of Wnt target genes by Wnt3a/Rspo2 (WR2) treatment of SK-N-BE(2)-C, SH-SY5Y and SK-N-AS cell lines relative to untreated (U) cells. A. Quantitative real-time PCR analysis of Wnt target genes was done on triplicate SK-N-BE(2)-C samples, and duplicate SH-SY-5Y and SK-N-AS samples. High induction is apparent for genes in the top two rows, and little or no induction of the genes in the third row. **B.** Immunoblotting showing effects of Wnt3a/Rspo2 treatment on c-MYC and MYCN at the protein level. Epidermal growth factor (EGF) was used as a positive control for proliferation studies and is also shown.

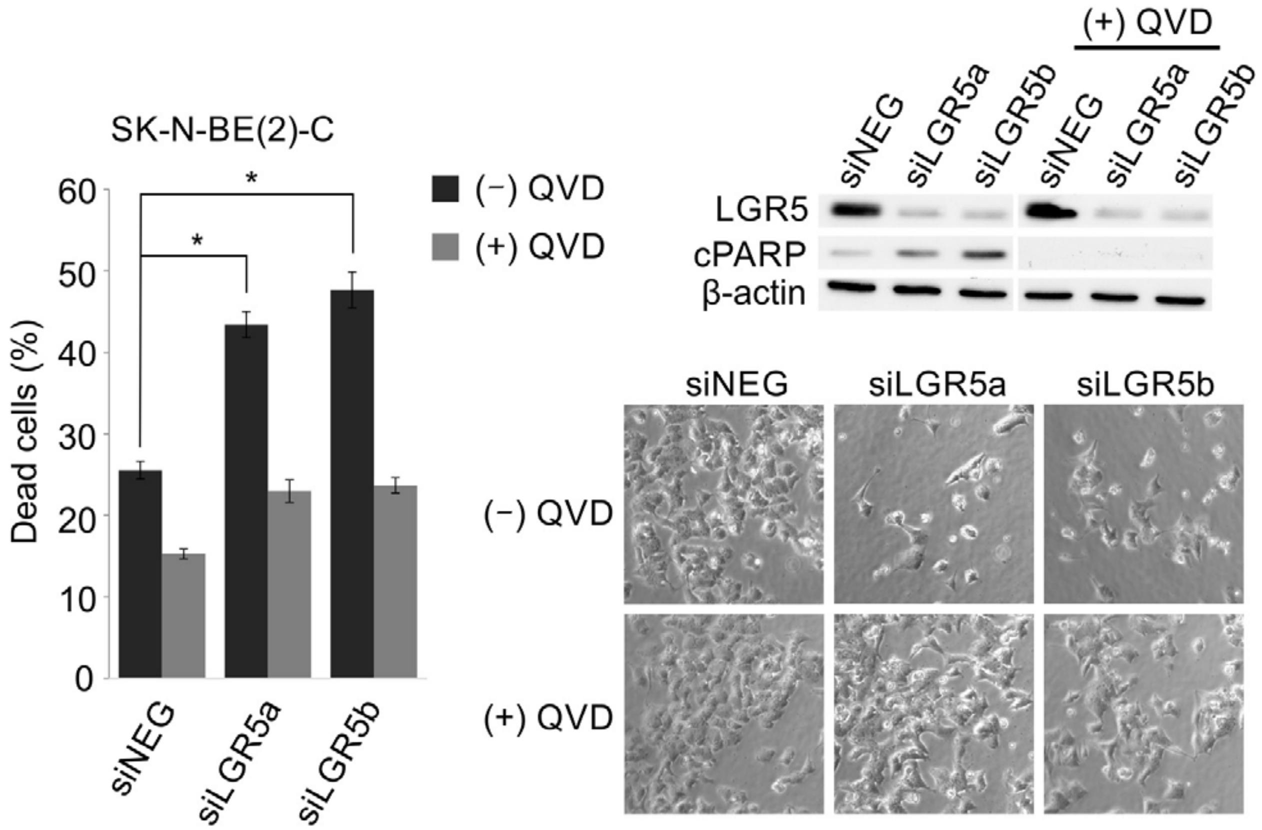

Supplementary Figure S3: Depletion of LGR5 induces apoptosis in SK-N-BE(2)-C cells. Knockdown of LGR5 with independent siRNAs induces apoptosis shown by cell-counts, increased cleaved PARP (cPARP), and rescue by the caspase inhibitor QVD. Asterisks denote $p < 0.05$, and assays are representative of at least three biological replicates.

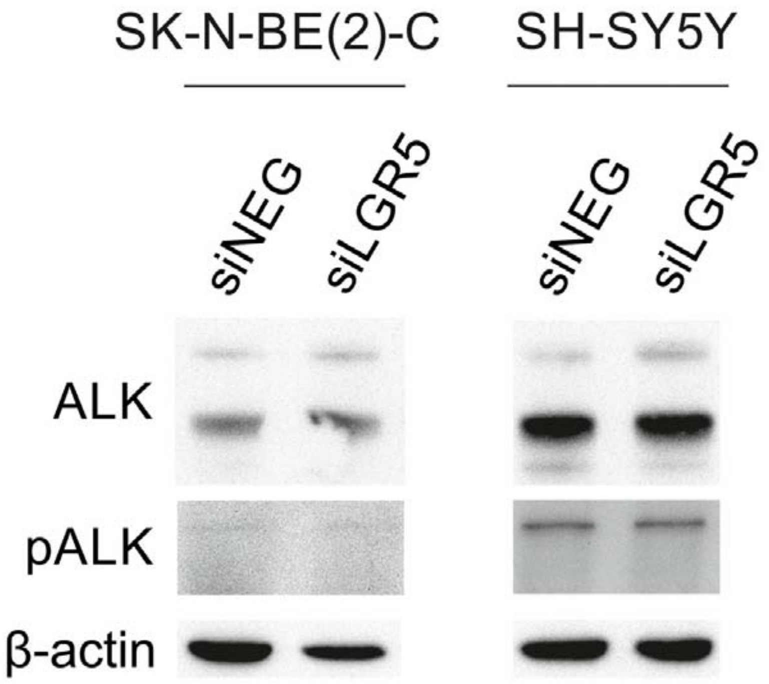

Supplementary Figure S4: ALK is not downregulated by LGR5 depletion. Immunoblotting demonstrates that ALK and phospho-ALK are not decreased after LGR5 knockdown in SH-SY5Y or SK-N-BE(2)-C cells. SK-N-AS cells do not express detectable ALK.

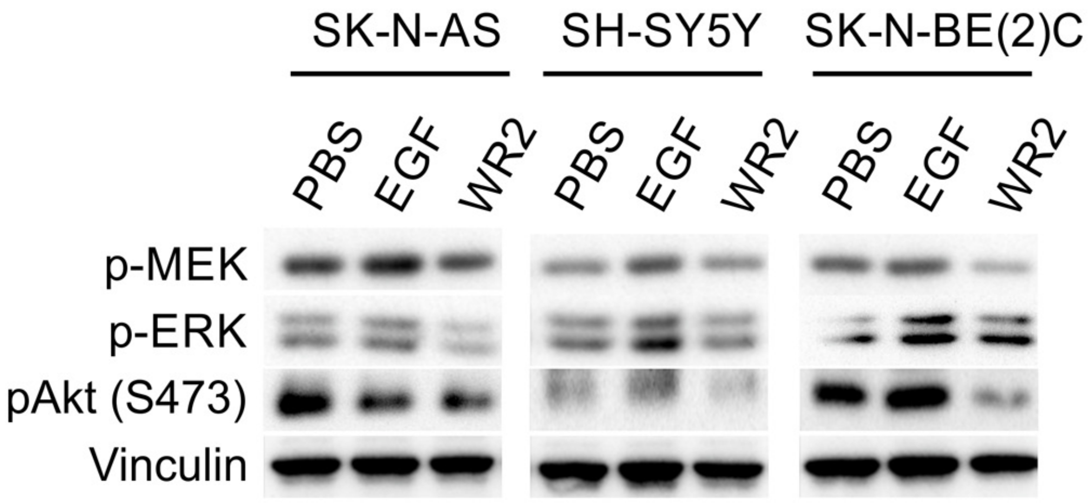

Supplementary Figure S5: Growth factor effects on phospho-MEK/ERK and phospho-Akt. Immunoblotting demonstrates that activated kinases are decreased after Wnt3a/Rspo2 treatment.

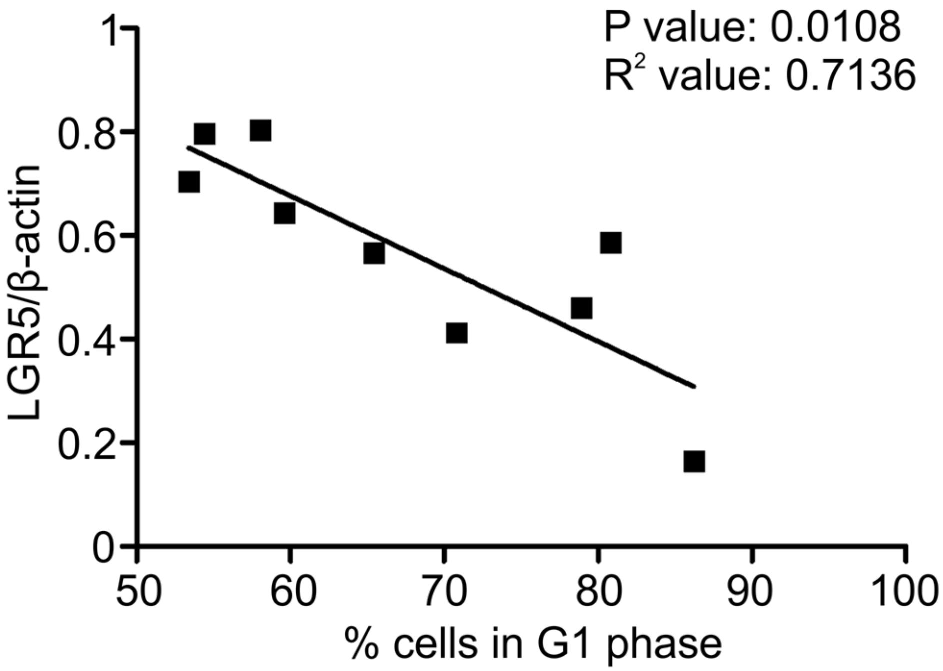

Supplementary Figure S6: LGR5 protein levels correlate with cell-cycle phase. Normalised LGR5 protein levels are plotted against percentage of cells in G1- or S-phase, as determined by flow cytometry.

Supplementary Table S1: Neuroblastoma cell lines` biological and genetic features

Cell Line	Primary site	metastatic site	stage	Type	MYCN status	ALK (1–3)	P53 (4–7)	NF1 protein expression (8, 9)
**SK-N-BE-(2)C**	unk.	bone marrow		I	Amp	WT	mut	
**GIMEN**	adrenal	Lymph node, bone marrow	4	S	NA			No
**IMR32**	abdomen	unknown	unk.	N	Amp		WT	Yes
**KELLY**				N	Amp	mut		Yes
**LAN1**	unk.	bone marrow	4	N	Amp	mut	mut	
**LAN5**	unk.	bone marrow	unk.		Amp	mut	WT	Yes
**LAN6**	adrenal	bone marrow, bone	4		NA		WT	
**NBL-S**	adrenal	none	3		NA	WT	WT	
**NGP**	unk.	bone marrow, lung	unk.	N	Amp	WT	WT	Yes
**SHEP**	thorax	bone marrow		S	NA		WT	
**SH-SY5Y**	thorax	bone marrow		N	NA	mut.	WT	Yes
**SK-N-AS**	adrenal	bone marrow	4	I	NA	WT	mut	Yes
**SKN-BE-(1)N**				N	Amp		WT	
**SMS-KAN**	pelvic	bone marrow, Lymphnode	4		Amp		WT	
**SMS-KANR**	pelvic	bone marow	4		Amp		WT	

The table displays the biological features of each cell line such as origin, primary site and metastatic site, stage and genetic and molecular features. unk.: unknown; N: neuroblastic cell type; I: Intermediate cell type; S: substrate-adherent cell type; Amp: amplified: NA: non-amplified; WT: wild type; mut: mutant

Supplementary Table S2: Neuroblastoma tumours` clinical and genetic features

Tumour	Diagnosis	Stage	MYCN status	Age at diagnosis (months)
**NB01**	NB	3	NA	1
**NB02**	NB	4	Amp	61
**NB03**	NB	4		66
**NB04**	NB	2	NA	3
**NB06**	NB	2	NA	14
**NB07**	NB	3	NA	35
**NB08**	NB	3	NA	4
**NB10**	NB	4	NA	60
**NB11**	NB	3		60
**NB13**	NB	4	NA	17
**NB14**	GNB	2	NA	23
**NB16**	NB in situ	–		New born
**NB17**	NB	4	NA	35
**NB18**	NB	4	NA	23
**NB19**	NB	3	NA	13
**NB20**	NB	4S	NA	7
**NB21**	NB	3	NA	7
**NB23**	GNB	2	NA	4
**NB25**	NB	4		47
**NB26**	NB	4	NA	149
**NB27**	GNB	–	NA	35
**NB30**	NB	2	NA	8
**NB31**	NB	4S	NA	6
**NB32**	NB	4	NA	55
**NB33**	GNB	1	NA	41
**NB35**	NB	1		8

The table displays some clinical features required by the International Neuroblastoma Staging System (INSS). NB: neuroblastoma; GNB: ganglioneuroblastoma; NA: non-amplified; Amp: amplified.

Supplementary Table S3 A: Antibodies used for protein expression analysis

Primary Antibody	Dilution
Akt (pan) (C67E7) Rabbit mAb (cell signaling technology 4691)	1:1000
Anti-Active-β-catenin (Anti-ABC) antibody, clone 8E7 (Millipore 05*665)	1:1000
Anti-c-Myc antibody [Y69] (abcam ab32072)	1:1000
Anti-GAPDH rabbit polyclonal antibody (abcam ab9485)	1:1000
Anti-Vinculin antibody [SPM227] (abcam ab18058)	1:1000
Anti-β-actin mouse monoclonal antibody (sigma, a5316)	1:1000
Cyclin E Antibody (HE12) (santa cruz biotech sc-247)	1:1000
GPR49 (LGR5) rabbit monoclonal antibody (abcam, ab75850)	1:1000
MEK1/2 (cell signaling technology 9122)	1:1000
Monoclonal anti-Lamin A/C produced in mouse (sigma SAB4200236)	1:1000
mTOR (7C10) Rabbit mAb (cell signaling technology 2983)	1:1000
N-Myc Antibody (B8.4.B) (santa cruz biotech sc-53993)	1:1000
p44/42 MAPK (Erk1/2) (cell signaling technology 9102)	1:1000
Phospho-Akt (Ser473) (D9E) XP® Rabbit mAb (cell signaling technology 4060)	1:1000
Phospho-Akt (Thr308) (D25E6) XP® Rabbit mAb (cell signaling technology 13038)	1:1000
Phospho-c-Raf (Ser338) (56A6) Rabbit mAb (cell signaling technology 9427)	1:1000
Phospho-MEK1/2 (Ser217/221) Antibody (cell signaling technology 9121)	1:1000
Phospho-p44/42 MAPK (Erk1/2) (Thr202/Tyr204) (20G11) Rabbit mAb (cell signaling technology 4376)	1:1000
Phospho-PDK1 (Ser241) (cell signaling technology 3061)	1:1000
Phospho-Rb (Ser807/811) (D20B12) XP® Rabbit mAb (cell signaling technology 8516)	1:1000
Rabbit monoclonal anti-Cleaved PARP antibody [E51] (abcam ab32064)	1:1000
Rictor (53A2) rabbit monoclonal antibody (cell signaling technology 2114	1:1000
Anti-Rabbit IgG peroxidase (Sigma, A6154)	1:5000
Anti-Mouse IgG peroxidase (Sigma, A4416)	1:5000

Primary and secondary antibodies used in all immunoblotting assays along with their respective recommended dilutions.

Supplementary Table S3 B: Confocal microscopy antibodies

Primary Antibody	Concentration	Secondary Antibody	dilution
**IgG1a monoclonal anti-β-catenin antibody (610154, BD Biosciences)**	10 ug/mL	Alexa Fluor 488 F(ab`)$_2$ fragment of Goat Anti-mouse IgG (H+L) (A-11017, Molecular Probes, Life Technologies)	1:500
**Purified mouse IgG1k isotype control (554121, BD Biosciences)**	10 ug/mL	Alexa Fluor 488 F(ab`)$_2$ fragment of Goat Anti-mouse IgG (H+L) (A-11017, Molecular Probes, Life Technologies)	1:500

The table displays the primary and secondary antibodies used for confocal immunofluorescence assay of neuroblastoma cell lines.

Supplementary Table S4 : Oligonucleotides used for real time PCR

Gene	Assay	Forward	Sequence (5'-3')	Reverse	Sequence (5'-3')
MYC	Real-time PCR	C-MYCRQF	AAGACTCCAGCGCCTTCTCTCCGT	C-MYCRQR	TGGGCTGTGAGGAGGTTTGCTGTG
MYCN	Real-time PCR	MYCNRQF1	CCTCAGTACCTCCGGAGAGGAC	MYCNRQR1	TGGGAAGGCATCGTTTGAGGATCA
BMP4	Real-time PCR	BMP-F	TGAGGGACGCGAGCCTGAGA	BMP-Rev	ACGGAATGGCTCCATAGGTCCC
AXIN2	Real-time PCR	HAXIN2rnaF	AGTGTGAGGTCCACGGAAAC	HAXIN2rnaR	ATGGACATGGAATCATCCGT
LEF1	Real-time PCR	LEF1-for	AATGAGAGCGAATGTCGTT	LEF1-rev	GCTGTCTTTCTTTCCGTGCT
TWIST	Real-time PCR	Twist rt-f	GCAGGGCCGGAGACCTAG	Twist rt-r	CTCCAGAGTCTCTAGACTGTC
TNFRSF19	Real-time PCR	TNFRSF19-qF	TGCTTGCCAGGATTTTATAGGAA	TNFRSF19-qRev	GACGCGATCTTCACGAGGTT
TBP	Real-time PCR	TBPRQF	GCCCGAAACGCCGAATAT	TBPRQR	CCGTGGTTCGTGGCTCTCT
JAG1	Real-time PCR	Jagged1rnaRQF	CGGGATTTGGTTAATGGTTATC	Jagged1rnaRQR	ATAGTCACTGGCACGGTTGTAGCAC
SOX2	Real-time PCR	F-SOX2-Q	CATGAACGGCTGGAGCAACG	R-SOX2-Q	TGCGAGTAGGACATGCTGTAGG
